# Neurocysticercosis in Oregon, 1995–2000

**DOI:** 10.3201/eid1003.030542

**Published:** 2004-03

**Authors:** John M. Townes, Christopher J. Hoffmann, Melvin A. Kohn

**Affiliations:** * Oregon Health and Science University, Portland, Oregon, USA; † Oregon Department of Human Services, Portland, Oregon, USA

**Keywords:** Neurocysticercosis, *Taenia solium*, parasitic diseases, hydrocephalus

## Abstract

The unexpected death of a teenager from neurocysticercosis prompted an investigation of this disease in Oregon. We found 89 hospitalizations, 43 newly diagnosed cases, and 6 deaths from 1995 to 2000. At least five cases occurred in persons who had not traveled or lived outside the United States. Enhanced surveillance for neurocysticercosis is warranted.

Neurocysticercosis, an infection of the central nervous system with the larval form of the pork tapeworm *Taenia solium*, causes substantial illness and death in developing countries. The disease has recently been increasingly recognized as a public health problem in the United States, primarily in southwestern states bordering Mexico (*1*). In nonborder states, however, neurocysticercosis may be an underrecognized problem.

In September 2000, Oregon’s Statewide Child Fatality Review Team reviewed the unexpected death of a 17-year-old girl due to neurocysticercosis; the diagnosis was made postmortem. We describe this case and present the results of the investigation of the epidemiology of neurocysticercosis in Oregon that followed.

## Case Report

**Figure 1:**
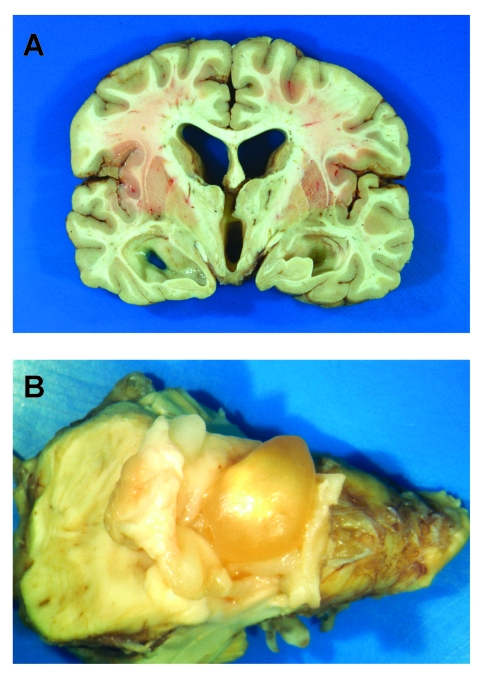
A) Coronal section of brain showing dilation of ventricles, flattening of the cerebral gyri, and uncal herniation. B) Intact cysticercus occupying the 4th ventricle

A previously healthy teenage girl, who had immigrated to Oregon from Mexico as an infant, sought care in January 2000 for progressively severe headaches of several months duration. After three office visits, “tension headaches” were diagnosed, and symptomatic therapy was prescribed. Neuroimaging was not performed. Several days later she was found dead at home in her bed. Autopsy showed no evidence of trauma, and results of a toxicology screening were negative. Examination of the brain showed obstructive hydrocephalus, bilateral uncal herniation, flattening of the cerebral gyri, and an intact cysticercus compressing the inferior 4th ventricle (Figure 1).

## The Study

We searched the Oregon Hospital Discharge Database to identify Oregon hospitals that had discharged patients with the ICD-9-CM code for neurocysticercosis (123.1) from January 1995 through December 2000 and requested the medical records of these patients. Available medical records were abstracted by using a standardized data collection instrument. We searched the Oregon death certificate database for additional neurocysticercosis deaths during the same period.

A case of neurocysticercosis was defined as any person with a hospital discharge code of 123.1 or death certificate diagnosis of neurocysticercosis during the study period, and a record of imaging studies or pathology reports consistent with neurocysticercosis. An incident case was defined as one for which no reference to any previous diagnosis of neurocysticercosis was found in the medical record. Incidence rates were calculated by using U.S. Census Bureau yearly population estimates for Oregon as the denominator (*2*,*3*). Data were analyzed with EpiInfo, version 6.04d (Centers for Disease Control and Prevention, Atlanta, GA).

**Figure 2:**
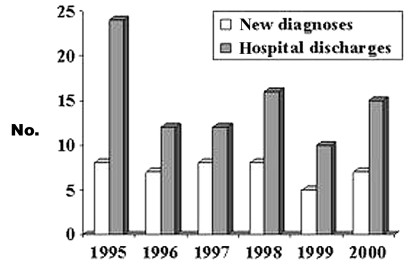
Number of neurocysticercosis hospital discharges and new diagnoses by year; Oregon, January 1995–December 2000.

We found 89 hospital discharges coded for neurocysticercosis during the study period among 18 hospitals in 10 counties. Medical records were made available for 76 (85%) of these hospitalizations. Review of these records confirmed the diagnosis of neurocysticercosis for 59 persons (17 were admitted more than once). Review of death certificates showed one additional nonhospitalized case-patient. Thus, including the death described above, we found 61 persons who met the case definition for neurocysticercosis; 43 (70%) of these were incident cases. Figure 2 shows neurocysticercosis hospital discharges and new diagnoses by year. The annual number of incident cases did not change significantly during the study period. The mean annual incidence rate from 1995 to 2000 was 0.2 per 100,000 general population and 3.1 per 100,000 Hispanic population.

Among the 61 confirmed patients, 40 (66%) were male, and 52 (85%) were Hispanic. The median age at the time of first hospitalization was 24 years (range 2 to 79 years); 13 (21%) were <18 years old. Occupation was recorded for 37 adult patients. Agriculture or other manual labor was the most common job type listed (20/37; 54%); 21% patients were unemployed or disabled; 40% of patients had no health insurance.

The country of birth was recorded for 57 persons. Of these, 41 (72%) were born in Mexico, 10 (18%) in the United States, 3 (5%) in Guatemala, and 1 (2%) each in Korea, Saudi Arabia, and an unspecified African country. Of the 10 neurocysticercosis patients born in the United States, 5 had never traveled outside the United States. Four of these five cases occurred among children (ages 3, 5, 5, and 12 years); their source of exposure to *Taenia* eggs was not documented. The source of exposure for the adult patient was presumed to be a household contact visiting from Mexico. Travel histories of four of the remaining U.S.-born patients included a Caribbean cruise by a retired pig farmer, extensive travel in Madagascar and Tanzania by a student, annual visits to Puerto Rico by an 11-year-old, and 1 week spent in Mexico City by a toddler. No information about travel was documented for one patient, a 3-year-old child.

The median duration of hospitalization was 3 days (range 1–30 days). Admission to an intensive care unit occurred in 21% of hospitalizations. Treatment included craniotomy with cyst removal (16%),ventriculoperitoneal shunting (12%), albendazole (18%), praziquantel (19%), anticonvulsive therapy (63%), and corticosteroids (49%).

Five persons died in addition to the patient described here. Two of these deaths also occurred unexpectedly before diagnosis; both resulted from acute hydrocephalus caused by an obstructing cyst on the 4th ventricle. Three patients who had been previously diagnosed with neurocysticercosis died. The causes of death in these patients were aspiration pneumonia after craniotomy and cyst removal, intracerebral hemorrhage, and hydrocephalus due to recurrent cerebrospinal fluid shunt failure. The median age at time of death was 33 years (range 17–73 years).

## Conclusions

This study demonstrates that neurocysticercosis causes substantial illness and death among Hispanic populations in Oregon. Many of those affected are young immigrants from Mexico without medical insurance, who are either unemployed or are working in agriculture or other manual labor. At least five cases appear to have been acquired in the United States. Our study did not address where disease transmission occurred for persons born outside of the United States; some of these cases could also have been acquired locally.

Several previous reports have documented the increasing recognition of neurocysticercosis in the United States, but the emphasis has been primarily on disease occurring in southwestern states bordering Mexico (*4*) and small outbreaks elsewhere (*5*–*7*). Previously, the only available information about the occurrence of neurocysticercosis in the Pacific Northwest was a study of seizure patients at 11 university-affiliated, urban emergency departments throughout the United States. In that study, cases were concentrated in southwestern states; four cases were found in Oregon during a 2-year period (*8*). The study underestimated the incidence of this disease in the Northwest, and perhaps other areas of the United States, by looking for cases only in selected urban emergency departments.

The average annual incidence of neurocysticercosis among Oregon’s Hispanic population found in our study is higher than that previously reported in Los Angeles County (1.6/100,000) (*9*) and in Mexico (0.8/100,000) (*10*). Low U.S. Census Bureau population estimates due to undercounting of Hispanic migrants could have resulted in a falsely elevated incidence rate. In addition, the higher observed incidence in Oregon may have been a result of greater case ascertainment because hospital discharge data were used rather than physician and laboratory reports. Nevertheless, because of our study design, we probably underestimated the true number of incident cases. Persons in outpatient clinics and emergency departments in whom neurocysticercosis had been diagnosed (who did not become hospitalized) were not counted in our study. We also did not include in the analysis of cases 15% of hospital discharge diagnoses that we could not confirm by chart review; if these had been confirmed as neurocysticercosis, then the true number of new cases may have been even higher. Although the Hispanic population in Oregon grew by an estimated 67% during the study period (*2*,*3*), the number of hospitalizations and new diagnoses did not increase. Whether this finding represents a true decrease in incidence or a shift in diagnosis and management of these cases to the outpatient setting, is unclear.

Neurocysticercosis has not previously been a reportable condition in Oregon, and no public health followup of the patients with locally acquired cases was performed to determine the source of these persons’ exposures to *Taenia* eggs. Previous serologic studies (*11*,*12*) would suggest that these patients acquired disease from household contact with a *T. solium* carrier. Early identification and public health followup of neurocysticercosis patients may lead to the recognition and treatment of tapeworm carriers, thereby preventing additional cases. However, since excretion of *Taenia* eggs is intermittent, direct parasitologic examination of stools is not a sensitive test (*11*). Collecting multiple stool samples from asymptomatic persons may also be a challenge. A serologic test for detection of taenaisis has been developed (*13*), but it is not yet commercially available. Public health providers need simple, inexpensive, and readily available techniques for rapidly identifying *Taenia* carriers in order to conduct optimal followup of cases and prevent additional cases.

The World Health Organization has estimated that more than 2 million persons are infected with adult tapeworms (*14*). As a result of increasing immigration and foreign travel, *T. solium* will likely continue to emerge as an important pathogen in the United States. Cysticercosis and taeniasis were designated as reportable conditions in Oregon in 2002. We recommend that public health surveillance activities more accurately define the incidence and risk factors for illness in Oregon, allow identification and treatment of tapeworm carriers, and provide epidemiologic and clinical data to physicians caring for patients in at-risk populations.
